# Enhancing Cancer Therapy with TLR7/8 Agonists: Applications in Vaccines and Combination Treatments

**DOI:** 10.3390/cancers17213582

**Published:** 2025-11-06

**Authors:** Jagannath Mondal, Swayam Prabha, Thomas S. Griffith, David Ferguson, Jayanth Panyam

**Affiliations:** 1 Department of Pharmaceutics, School of Pharmacy, University of Washington, 1959 NE Pacific Street, Seattle, WA 98195, USA; jmondal@uw.edu (J.M.); sprabha@uw.edu (S.P.); 2 Department of Urology, Medical School, University of Minnesota, Minneapolis, MN 55455, USA; tgriffit@umn.edu; 3 Department of Medicinal Chemistry, College of Pharmacy, University of Minnesota, Minneapolis, MN 55455, USA; ferguson@umn.edu

**Keywords:** Toll-like receptors, TLR 7/8 agonists, immunotherapy, combination therapy, cancer vaccines

## Abstract

Targeting Toll-like receptors 7 and 8 (TLR7/8) has emerged as a promising strategy in cancer immunotherapy. TLR7/8 agonists activate robust Th1-type immune responses and bridge innate and adaptive immunity. Further, TLR7/8 agonists can serve as valuable adjuncts to conventional therapies, such as chemotherapy and radiotherapy, enhancing efficacy while reducing adverse effects. Their integration into combination regimens for cancer offers a dual advantage: amplifying antitumor immunity and reducing tumor burden. Notably, the incorporation of TLR7/8 agonists into cancer vaccine platforms has yielded encouraging results in preclinical models and is advancing toward clinical application. This review highlights the mechanisms of action, therapeutic potential, and recent progress in the development of TLR7/8 agonist-based strategies for cancer treatment. We also discuss ongoing clinical evaluations and the rationale for combining these agents with existing modalities to enable more effective, personalized, and accessible cancer therapies.

## Simple Summary

Targeting immune system proteins called TLR7 and TLR8 is showing promise in cancer treatment. These proteins help activate a strong immune response that can detect and attack cancer cells. Drugs that stimulate TLR7/8 not only boost immunity but also enhance the effects of traditional treatments like chemotherapy, radiation, and phototherapy-potentially improving therapy outcomes and reducing side effects. TLR7/8 agonists are also being used in cancer vaccines, which train the body to fight cancer more precisely. Early results in lab studies have been encouraging, and clinical trials are now testing their effectiveness in people. This approach offers a powerful combination: it strengthens the body’s natural defenses while working alongside existing therapies to reduce tumor size. Using TLR7/8 agonist-based drugs in personalized cancer treatments may improve effectiveness, reduce side effects, and increase accessibility to more patients.

## 1. Introduction

Cancer remains a leading cause of mortality worldwide, accounting for approximately one in six deaths. Despite advances in conventional treatments such as chemotherapy, radiotherapy, and surgical resection, these approaches are often associated with significant side effects and limited long-term efficacy. As a result, there continues to be a need for more sophisticated and targeted therapeutic strategies to treat cancer.

Cancer immunotherapy has emerged as a transformative approach, harnessing the body’s immune system to recognize and eliminate malignant cells [1,2,3]. This strategy offers several advantages over traditional treatments, including enhanced specificity, potential for long-lasting immune memory [4], broader applicability across cancer types [5], potential for additive or synergistic effects [6], reduced resistance [7], and opportunities for personalized medicine [8]. Notable examples include immune checkpoint inhibitors (e.g., anti-CTLA-4, anti-PD-1/PD-L1 antibodies) [9,10,11], chimeric antigen receptor (CAR) T-cell therapy [12,13,14], and monoclonal antibody-based treatments [15], all of which have significantly improved patient outcomes in various cancers.

Antigen-presenting cells (APCs), such as dendritic cells (DCs) and macrophages, play a central role in initiating and shaping antitumor immune responses by presenting tumor-associated antigens to T cells [16,17]. The activation of cytotoxic T lymphocytes (CTLs) and natural killer (NK) cells is also critical for effective tumor clearance [18,19]. For instance, mature DCs exhibit enhanced immunostimulatory capacity compared to their immature counterparts, contributing to more robust antitumor immunity [20].

Despite these advances, challenges remain in achieving durable responses across all patient populations. One key area of interest is the use of agents that selectively target pattern recognition receptors (PRRs), particularly Toll-like receptors (TLRs), which are expressed on both immune and tumor cells [21]. TLRs serve as a bridge between innate and adaptive immunity and play a pivotal role in immune surveillance and activation [22]. Multiple Toll-like receptors (TLRs) have been explored as immunotherapeutic targets. Among them, TLR7 and TLR8 have attracted particular interest due to their ability to be activated by small molecules. Activation of TLR7/8 stimulates the production of NF-κB-mediated pro-inflammatory cytokines and chemokines [23]. This, in turn, triggers robust innate and adaptive immune responses, enhancing APC function and cytotoxic T cell priming-both essential for effective cancer vaccination. Furthermore, TLR7/8 activation promotes tumor cell apoptosis and strengthens antitumor immunity [24]. TLR7/8 agonists also modulate immunosuppressive myeloid populations and improve the efficacy of immune checkpoint inhibitors, underscoring their translational relevance in cancer immunotherapy. Several small-molecule TLR7/8 agonists, including imiquimod, motolimod, vesatolimod, and DN052, have advanced into clinical investigation, providing a strong foundation of preclinical and clinical evidence for further evaluation.

**Figure 1:**
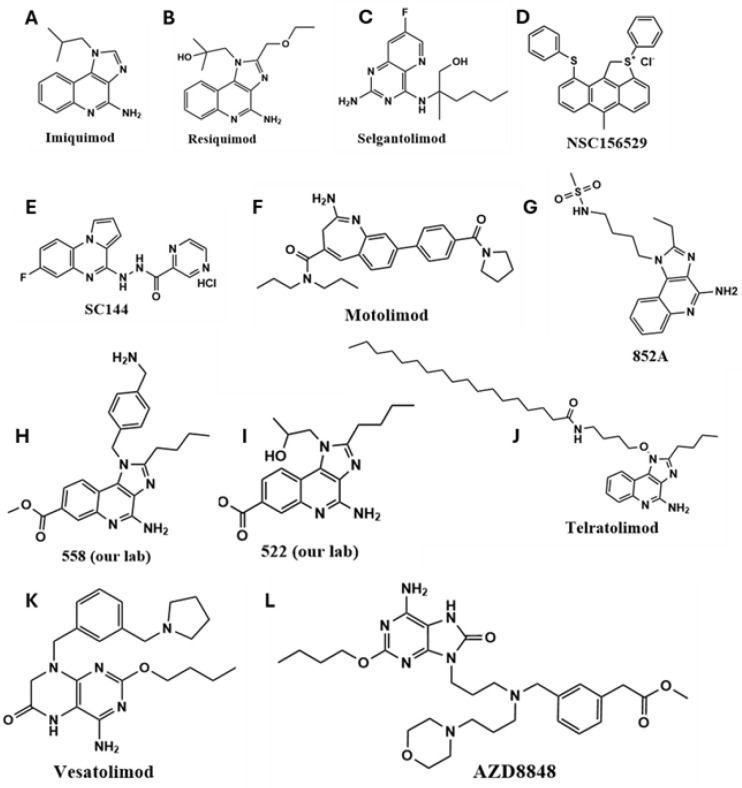
The chemical structures of synthetic TLR7/8 agonists and their analogs have been used for preclinical and clinical translation. (**A**) IMQ, (**B**) Resiquimod, (**C**) Selgantolimod, (**D**) NSC156529, (**E**) SC144, (**F**) Motolimod, (**G**) 852A, (**H**) 558, (**I**) 522, (**J**) Telratolimod, (**K**) Vesatolimod and (**L**) AZD8848.

Recent developments have focused on small-molecule and nucleic acid-based TLR7/8 agonists [22,25], which are being evaluated in both preclinical and clinical settings. Pyrido [3,2-d] pyrimidine-derived small molecules have been utilized in the design of TLR agonists [26]. Compounds such as imiquimod (R837; IMQ), the first FDA-approved TLR7 agonist for basal cell carcinoma [27], and its more potent derivative resiquimod (R848), have demonstrated promising immunostimulatory effects [28,29,30]. Other agents, such as 852-A, have shown efficacy in hematological malignancies by enhancing adaptive immune responses [31]. Other TLR7/8 agonists that have been explored for cancer treatment are shown in Figure 1.

Although TLR agonists have demonstrated efficacy as monotherapies, they are increasingly being integrated into combination therapies as immune adjuvants [32]. For example, the combination of oxaliplatin with R848 significantly enhanced antitumor effects in colorectal cancer models [33], underscoring the potential of TLR7/8 agonists to boost the efficacy of conventional treatments. Several therapeutic modalities-including chemotherapy, radiation therapy, and phototherapy-induce tumor cell death but often do not generate durable immune responses. Because these treatments promote the release of tumor antigens, combining them with TLR7/8 agonists can result in the activation of antigen-specific immune response and long-term antitumor immunity. This combinatorial approach may also help overcome tumor immune evasion by enabling the immune system to better recognize and eliminate tumor cells. Ongoing research is focused on optimizing these combination strategies to maximize therapeutic efficacy while minimizing adverse effects.

This review provides a comprehensive overview of TLR7/8 agonist-based strategies in cancer immunotherapy. We discuss their mechanisms of action, structural diversity, and immunological effects, as well as their integration into cancer vaccines and combination therapies. We also highlight recent advances in preclinical and clinical development and address the challenges and safety considerations associated with TLR7/8 agonist-based treatments. While TLR-mediated immunotherapy has shown remarkable promise, further investigation is essential to fully realize its clinical potential and ensure safe, effective application in cancer care.

## 2. Search Strategy and Selection Criteria

This comprehensive review employed a strategic literature search to ensure broad coverage of studies investigating TLR7/8 agonist-based cancer vaccines and combination therapies. The search was conducted across electronic databases, including PubMed, Scopus, and Web of Science, targeting relevant research and review articles published between 2015 and 2025. Keywords used in the search included: TLR7 agonist, TLR8 agonist, TLR7/8 agonist, TLR7/8-based cancer vaccine, TLR7/8-based cancer immunotherapy, TLR7/8-based combination treatment, and TLR7/8-based clinical trials. Only peer-reviewed articles published in English were selected to highlight recent advancements in this field. Studies were included if they provided preclinical or clinical insights into the immunomodulatory agents, mechanisms of action, delivery strategies, or therapeutic efficacy of TLR7/8 agonists in cancer vaccination or combination approaches. Additional references were identified through cross-referencing the bibliographies of selected research and review articles.

## 3. Mechanisms of Activation and Downstream Signaling of the TLR Family

The innate immune system serves as the body’s first line of defense, providing a rapid response to injury or pathogen invasion and priming the adaptive immune system for a more specific, long-term response [34]. This early immune activation is mediated by pattern recognition receptors (PRRs), which detect pathogen-associated molecular patterns (PAMPs) and, in some cases, endogenous damage-associated molecular patterns (DAMPs), including tumor-derived antigens [35].

Several classes of PRRs have been identified based on their structural domains and cellular localization. These include Toll-like receptors (TLRs), nucleotide-binding oligomerization domain (NOD)-like receptors (NLRs), C-type lectin receptors (CLRs), retinoic acid-inducible gene-I (RIG-I)-like receptors (RLRs), and absent in melanoma 2 (AIM2)-like receptors (ALRs) [36,37]. Among these, TLRs are particularly important in modulating immune responses and linking innate and adaptive immunity [38]. To date, ten human TLRs have been identified. TLR1, TLR2, TLR4, TLR5, TLR6, and TLR10 are expressed on the plasma membrane, where they detect extracellular signals. In contrast, TLR3, TLR7, TLR8, and TLR9 are localized to intracellular compartments such as endosomes and the endoplasmic reticulum, where they recognize nucleic acid-based ligands [39]. Structurally, TLRs are composed of three main domains: An extracellular domain rich in leucine-rich repeats (LRRs), typically arranged in a horseshoe-like shape, which is responsible for ligand recognition. Each LRR consists of a β-strand and an α-helix connected by loops, spanning 24–29 amino acids. Secondly, a transmembrane domain anchors the receptor in the membrane. And finally, an intracellular Toll/IL-1 receptor (TIR) domain, which initiates downstream signaling upon receptor dimerization [40]. The dimerization is needed to activate TIR and initiate three transcriptional factors: AP-1, NF-kB, and IRF7 [41,42,43].

TLR family members vary across species. Humans possess 10 functional TLRs (TLR1–TLR10), whereas mice have 12 (TLR1–TLR12) [38]. Interestingly, the core structural domains and signaling pathways of TLRs are highly conserved across species [44]. In humans, TLR genes are distributed non-randomly across chromosomes: TLR1, TLR2, TLR3, TLR6, and TLR10 are located on chromosome 4; TLR4 on chromosome 9; TLR5 on chromosome 1; TLR7 and TLR8 on the X chromosome; and TLR9 on chromosome 3. In mice, TLR1, TLR2, and TLR6 are found on chromosome 4; TLR3 and TLR4 on chromosome 5; TLR7 and TLR8 on the X chromosome; TLR9 on chromosome 9; and TLR11–TLR13 on chromosome 14 [45].

### 3.1. Activation and Signaling of TLR 7/8 Ligand

**Figure 2:**
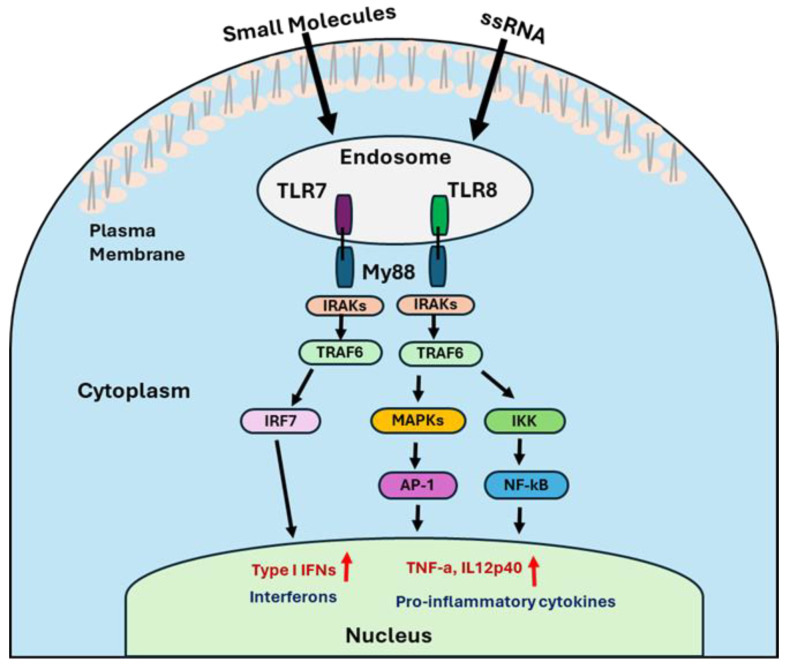
The illustration represents the mechanistic signaling pathways of TLR7/8 agonists.

Upon ligand binding, TLR7 and TLR8 undergo conformational changes that promote dimerization and recruitment of the adaptor protein MyD88 (myeloid differentiation primary response gene 88) via its C-terminal TIR domain [46]. The N-terminal death domain of MyD88 then recruits IL-1 receptor-associated kinases (IRAK4 and IRAK1) [47], forming a signaling complex that activates tumor necrosis factor receptor-associated factor 6 (TRAF6) [48]. TRAF6 subsequently activates multiple downstream pathways, including the formation of the IκB kinase (IKK) complex, activation of the mitogen-activated protein kinases (MAPK) family, and recruitment of IFN regulatory factors (IRF7). Then, IKK complex leads to the nuclear translocation of NF-κB, a key transcription factor involved in inflammation and immune activation. Similarly, the MAPK pathway, which activates transcription factors such as cyclic AMP response element-binding protein (CREB) and activator protein 1 (AP-1), promotes the production of pro-inflammatory cytokines like IL-6, IL-12p70, and TNF-α. Activation of the IRF7 pathway leads to the expression of type I interferons, including IFN-α and IFN-β (Figure 2) [22,49,50].

These signaling cascades collectively lead to the production of inflammatory cytokines and type I interferons, thereby enhancing both innate and adaptive immune responses. The ability of TLR7/8 to activate multiple immune pathways makes them attractive targets for immunotherapy, particularly in the context of cancer, where robust immune activation is essential for effective tumor clearance. Interestingly, we have identified that our lab-synthesized TLR7/8 agonist, 558, could produce type II IFN, in addition to type I IFNs. Global proteomics analysis was performed to investigate the mechanisms underlying the production of type II IFNs. Moreover, this study demonstrated that 558 synergistically activated STING and inflammasome pathways to its effect on TLR7/8 signaling on DC maturation [51].

### 3.2. Biomarkers for TLR7/8-Based Therapies

Identifying predictive biomarkers within the tumor microenvironment is essential for guiding cancer immunotherapy and improving patient outcomes. PD-L1 expression on tumor cells, for example, has been correlated with enhanced responses to anti-PD-1/PD-L1 therapies, although its predictive accuracy remains limited due to modest sensitivity and specificity [52,53]. Similarly, elevated tumor mutational burden (TMB) and inflammatory gene-expression profiles (GEPs) have shown promise as predictive indicators. However, a meta-analysis revealed that multiplex immunohistochemistry/immunofluorescence (mIHC/IF) outperformed these individual markers in predicting treatment response [54].

Tumor-infiltrating lymphocytes (TILs), particularly their density and phenotypic characteristics, have emerged as valuable biomarkers across various cancer types [55,56]. Myeloid cell signatures are also gaining attention for their potential to predict responses to immune checkpoint inhibitors, though their clinical application remains challenging [57]. Recently, TLRs, especially TLR4 and TLR7, have been investigated as prognostic biomarkers in the context of vaccine-based therapies. Meta-analyses suggest that high expression of TLR4 or TLR7 may be associated with poorer survival across multiple cancers [58,59]. Interestingly, one study reported that elevated TLR expression combined with high TIL density predicted improved outcomes in colorectal cancer [60].

Despite these promising findings, the clinical utility of these biomarkers in vaccine-based immunotherapy remains constrained by the lack of standardized thresholds, multicenter validation, and robust prospective data. Continued research is needed to refine biomarker selection and validation, enabling more personalized and effective vaccine strategies in cancer treatment.

## 4. Challenges Associated with TLR7/8 Agonists

A major challenge with the use of TLR7/8 agonists is the variability in patient response. Identifying individuals who are most likely to benefit from TLR-based therapies is critical. For example, two independent studies found that combining the TLR8 agonist motolimod with chemotherapeutic drugs such as 5-FU, DOX, and platinum did not improve overall survival across all patients. However, a predefined subpopulation exhibited significantly better progression-free and overall survival outcomes [61,62]. This highlights the importance of stratifying patients based on gene expression profiles, biomarker levels, and local immune responses to optimize treatment efficacy.

Systemic administration of TLR7/8 agonists can also lead to excessive cytokine release, resulting in adverse effects such as fever, hypotension, and, in severe cases, cytokine release syndrome. Natalie et al. reported that systemic delivery of R848 in mice caused brain swelling [63]. Another study showed that R848 induced acute sickness behaviors, including hypophagia, weight loss, and reduced locomotor activity, along with elevated pro-inflammatory gene expression in the central nervous system [64].

In addition to systemic toxicity, poor tumor penetration can limit therapeutic efficacy. To address this, novel delivery systems are being developed to enhance tumor targeting and minimize off-target effects. For instance, the lipophilic TLR7/8 agonist MEDI9197 demonstrated improved retention at the injection site, underscoring the challenges of systemic transport and tumor localization [65]. Another concern is immune desensitization following repeated exposure to TLR agonists. Prolonged treatment with R848 induces a refractory state in microglia, characterized by diminished pro-inflammatory responses and reduced sickness behavior in mice [64]. However, Bourquin et al. demonstrated that adjusting the dosing schedule, using repeated low-dose R848 injections spaced five days apart, significantly reduced tumor burden compared to a single high-dose regimen. This suggests that optimizing dosage and timing may help overcome TLR tolerance [66].

## 5. Combination Therapies Involving TLR 7/8 Agonists

To further enhance therapeutic outcomes and prevent immune escape, TLR7/8 agonists are increasingly being combined with other treatment modalities, including immune checkpoint inhibitors, chemotherapy, radiation therapy, and phototherapy. These combinatorial strategies aim to synergistically activate the immune system while directly targeting tumor cells, offering a more comprehensive and durable approach to cancer treatment.

### 5.1. TLR7/8 Agonist Combined with Other Immunostimulators

Various tumor-driven mechanisms can impair CD8^+^ T cells’ activation and function, limiting the effectiveness of TLR7/8 agonists. To enhance therapeutic outcomes, various immunostimulatory agents have been co-administered with TLR7/8 agonists.

**Figure 3:**
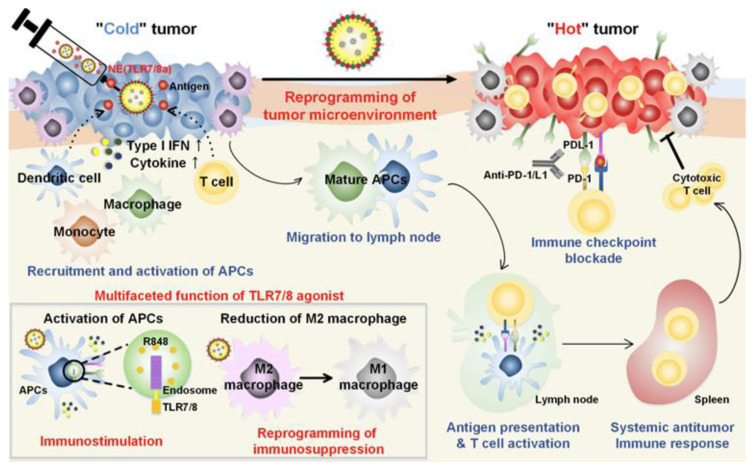
Schematic representation of the mechanisms of combining TLR7/8 agonist and checkpoint inhibitor for cancer immunotherapy. Adapted with permission from [68]. Copyright © 2019, American Chemical Society. IFN-I: type I IFNs, APCs: antigen-presenting cells, R848: an immune adjuvant resiquimod.

For instance, Kim et al. demonstrated that combining a tyrosine kinase inhibitor (sunitinib) with a TLR7/8 agonist (compound 522) in a nanoparticle-based vaccine significantly reduced the presence of myeloid-derived suppressor cells (MDSCs) and regulatory T cells (Tregs) in tumors [67]. The addition of anti-PD-L1 antibodies further alleviated CD8^+^ T cell exhaustion. This triple combination therapy not only reduced immunosuppressive cell populations but also activated CD8^+^ T cells and promoted memory responses. In another study, a nanoemulsion (NE) formulation of R848 was used to overcome tumor-induced immunosuppression and stimulate T cell-mediated responses. Mice treated with NE(R848) showed significantly reduced tumor progression and improved survival compared to those receiving free drug. When NE(R848) was combined with ovalbumin (OVA) and anti-PD-L1, the treatment led to enhanced T cell infiltration, elevated IFN-γ secretion, and superior tumor control. A tumor rechallenge experiment confirmed the induction of long-term antitumor memory. The simplicity of NE(R848) fabrication, its lyophilization capability, and the use of clinically safe components make it a promising platform for cancer immunotherapy and infectious disease management (Figure 3) [68].

Ni et al. developed a nanoscale metal–organic framework (nMOF) system, Hf-DBP-modified to co-deliver a hydrophilic anti-CD47 antibody (αCD47) and a hydrophobic TLR7/8 agonist (IMD) [69]. This platform enabled radiotherapy–radiodynamic therapy and reprogrammed the tumor microenvironment. IMD repolarized M2 macrophages to the pro-inflammatory M1 phenotype, while αCD47 promoted phagocytosis by blocking the “don’t eat me” signal. When combined with anti-PD-L1, this strategy eliminated both primary and distant tumors in a bilateral colorectal cancer model, highlighting the potential of MOFs for delivering immune adjuvants in macrophage-targeted therapies.

Moreover, Jiang et al. developed cancer cell membrane-coated MOFs to deliver 3M-052 (M) and epigenetic inhibitors, such as BRD4, for the treatment of TNBC [70]. This treatment modality resulted in significant recruitment of DCs with long-term antitumor immune response. Furthermore, this study demonstrated increased lymphocyte infiltration and ICD along with reduced Tregs. To address systemic toxicity associated with TLR agonists, Smith et al. developed PEG–PLA nanoparticles functionalized with TLR7/8 agonists [71]. These self-assembling nanoparticles improved pharmacokinetics, reduced systemic exposure, and enhanced tumor-specific delivery of the agonists. In vivo studies showed elevated serum IFN-α levels and improved therapeutic efficacy when combined with anti-PD-L1, resulting in reduced tumor growth and extended survival in a murine colon adenocarcinoma model.

**Figure 4:**
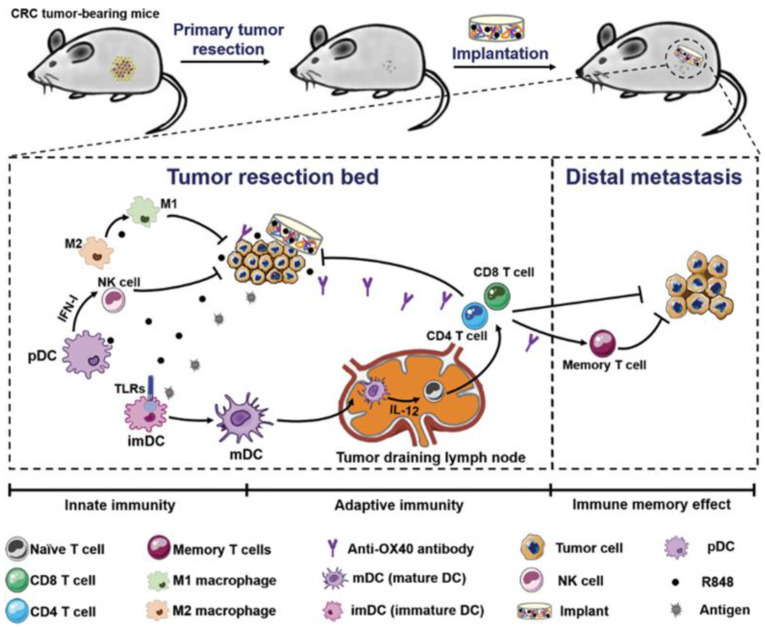
Schematic presentation of treatment strategy and implementation of biopolymers co-encapsulating R848 and anti-OX40 antibody, and possible mechanisms in immune responses after the treatments in colorectal cancer. Reprinted with permission from [72]. Copyright © 2020, John Wiley and Sons. TLRs: Toll-like receptors, IFN-I: type I IFNs, imDC: immature dendritic cells, mDC: mature dendritic cells, R848: an immune adjuvant resiquimod.

Postoperative tumor recurrence and metastasis remain major clinical challenges. Ji et al. addressed this by designing a biopolymer implant composed of 4-arm PEG-NH_2_ and oxidized dextran (ODEX) to co-deliver R848 and anti-OX40 antibodies following colorectal cancer surgery. This implant eradicated residual tumors for up to 150 days, inhibited distant tumor growth, and induced durable immune memory. Early immune responses included increased NK cell infiltration and DC activation, followed by robust T cell infiltration (Figure 4) [72].

Another delivery strategy was implemented by Kang et al. to treat ovarian cancer. The group developed large anionic liposomes (RSQLP) for intraperitoneal delivery of R848. This system specifically targeted tumor-associated macrophages (TAMs), repolarizing them to the M1 phenotype. It also increased tumor-infiltrating CD8^+^ and CD4^+^ T cells. When combined with anti-PD-L1, the treatment led to complete tumor rejection for up to 250 days, indicating long-term antitumor immunity [73].

Huang et al. designed ROS-responsive mesoporous nanoparticles (MSN@TheraVac) conjugated with anti-PD-L1 to co-deliver nucleosome-binding protein 1 (HMGN1) and R848 for colon cancer treatment [74]. This system enabled sequential release: rapid αPD-L1 to prevent T cell exhaustion, followed by HMGN1 and R848 to mature DCs. The treatment eliminated tumors in CT26-bearing mice and enhanced tumor-specific immune responses in draining lymph nodes.

Finally, Bhatnagar et al. implemented a multi-adjuvant strategy combining a TLR7/8 agonist with a STING agonist (DMXAA) and OVA antigen [75]. This combination matured DCs in tumors, spleen, and lymph nodes, and significantly increased antigen-specific CD8^+^ T cell and NK cell responses. The triple therapy reduced tumor burden and improved survival compared to other treatment groups.

Antibody–drug conjugates (ADCs) represent a targeted therapeutic strategy designed to deliver drugs directly to tumor sites, minimizing systemic toxicity. Among these, TLR7/8 agonist–antibody conjugates have shown promising results in preclinical models, including enhanced production of pro-inflammatory cytokines and improved therapeutic efficacy in mice [76,77,78,79]. However, translation into clinical trials has encountered significant challenges, particularly related to neuroinflammation and cytokine release syndrome, which compromise both safety and therapeutic effectiveness. To address these issues, several studies have investigated species-specific differences that may affect the translatability of preclinical findings to human applications [80,81].

For instance, Sega et al. employed mouse-derived antibodies conjugated with a TLR7 agonist to treat the CT26 syngeneic colon carcinoma model [82]. Their findings revealed significantly higher accumulation of the TLR7 agonist at the tumor site compared to free drug administration. The ADCs also demonstrated consistent processing via antibody-dependent cellular cytotoxicity (ADCC), resulting in sustained activation of myeloid cells within the tumor microenvironment (TME). In another study, a tumor-targeting LIV1 antibody was conjugated with TLR7/8 agonists to assess antitumor responses [83]. This approach enhanced inflammation and T cell recruitment in the TME more effectively than free drug treatment, leading to improved myeloid cell activation, antitumor efficacy, and tolerability. Additionally, a vaccine platform incorporating neoantigen–TLR7/8 agonist conjugates was developed to stimulate anticancer T cell responses. These constructs significantly boosted T cell activity against tumor antigens and offered a versatile framework for co-delivering peptides and adjuvants in cancer immunotherapy [84].

Various combination strategies involving antibodies, inhibitors, or STING agonists alongside TLR7/8 agonists have been employed to enhance therapeutic efficacy. These combinations have been delivered using platforms such as liposomes, nanoemulsions, metal–organic frameworks (MOFs), or as free drugs. Studies have demonstrated that these approaches elicit stronger antitumor immune responses compared to treatment with TLR7/8 agonists alone. While the addition of antibodies has shown promising therapeutic outcomes, a thorough evaluation of antibody-associated toxicities is essential before advancing to clinical applications. Moreover, testing these combinations across a broader range of cancer cell lines—rather than limiting evaluations to one or two—could help identify cancer-specific requirements and improve the translatability of these therapies to clinical settings.

### 5.2. Combination with Chemotherapy

Chemotherapy remains a cornerstone of cancer treatment, with drugs such as paclitaxel, doxorubicin, and cisplatin widely used due to their ability to induce immunogenic cell death (ICD) [70,85,86,87]. Combining chemotherapeutic agents with TLR7/8 agonists has shown enhanced therapeutic efficacy by simultaneously promoting tumor cell death and immune activation [88]. For example, co-administration of oxaliplatin with liposome-encapsulated R848 significantly increased CD8^+^ T cell infiltration and markedly reduced CT26 murine colorectal tumor growth compared to free drug treatments. This combination demonstrated the potential of liposomal R848 to enhance immune activation in colorectal cancer [89].

**Figure 5:**
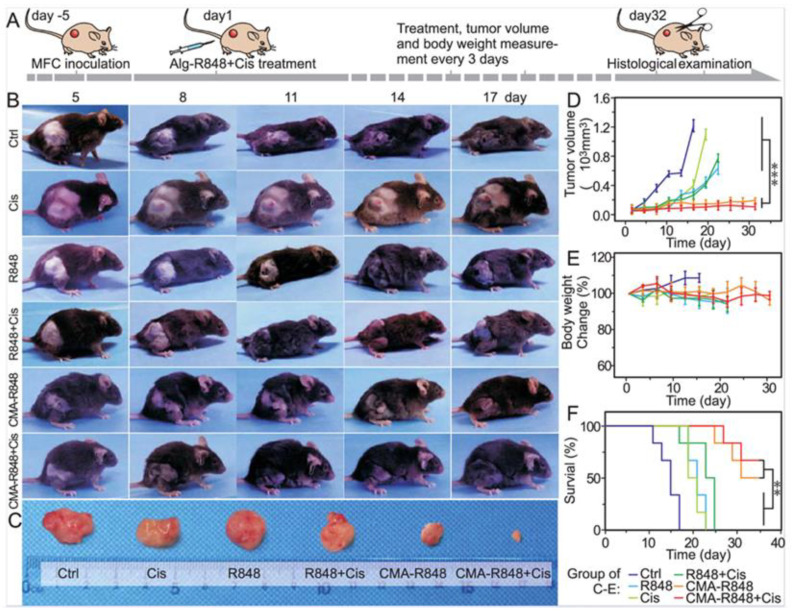
Evaluation of antitumor efficacy of CMA-R848 and cisplatin treatment in vivo studies on GC model mice. (**A**) The illustration of tumor establishment in the mouse model and the schedule for subsequent treatments with therapeutics. (**B**,**C**) The images of the Tumor volume following various treatments. (**D**,**E**) Tumor volume changes with various treatments. (**F**) Survival rate of the mice with individual treatments. *n* = 6, The statistical analysis was determined by one-way ANOVA test, ** *p* < 0.01, *** *p* < 0.001. Adapted with permission from [90]. Copyright © 2023, American Chemical Society. CMA: carboxymethylated alginate, R848: an immune adjuvant resiquimod.

In another study, micelles were engineered by conjugating hydrophilic carboxymethylated alginate with hydrophobic R848, forming CMA-R848 for gastric cancer therapy. To further improve efficacy, cisplatin was co-administered with the R848-loaded micelles. Upon reaching the mildly acidic tumor microenvironment, ester bonds in the micelles cleaved, releasing R848 locally. CMA-R848 treatment activated bone marrow-derived dendritic cells (BMDCs) and elevated IL-6 and TNF-α levels. Additionally, M2 macrophages were repolarized to the M1 phenotype, and IL-10 levels decreased while IL-12 increased. The combination of CMA-R848 and cisplatin significantly reduced tumor burden and enhanced CD4^+^ and CD8^+^ T cell populations, suggesting a shift from a “cold” to a “hot” tumor microenvironment (Figure 5) [90].

Jianqin et al. developed matrix metalloproteinase-2 (MMP-2)-sensitive hydrogels for chemo-immunotherapy in metastatic breast cancer [91]. These hydrogels incorporated nuclear-targeted tetrahedral DNA nanostructures and disulfide-crosslinked polyethylenimine, loaded with doxorubicin (DOX) and R837. The nanoparticles effectively penetrated tumor tissues and promoted tumor-associated antigen (TAA) release. In vitro, the combination therapy significantly increased secretion of IL-6, IL-12p70, and TNF-α. In vivo, CD8^+^ T cell populations rose to 5.52%, indicating a strong cytotoxic immune response.

Another study utilized a thermoresponsive hydrogel vaccine for breast cancer. Pluronic F127 polymers formed the hydrogel matrix, embedding polydopamine nanoparticles coated with hyaluronic acid. IQ (a TLR7 agonist) was combined with DOX to enhance tumor-specific immune activation through memory T cell proliferation and dendritic cell (DC) maturation. This injectable hydrogel platform significantly slowed tumor growth and eliminated tumors within 7–21 days. Treated mice exhibited elevated memory T cell and DC responses and extended survival to two months, compared to one month in untreated controls [92].

For aggressive melanoma, injectable hydrogels composed of polyethylene glycol thiol and poly(ethylene glycol) diacrylate were used to co-deliver R837 and DOX. This strategy minimized systemic toxicity and improved therapeutic outcomes. The combination treatment suppressed melanoma growth both in vitro and in vivo, induced ICD, activated DCs, and promoted M1 macrophage polarization. Cytokines such as IFN-γ and TNF-α were significantly elevated in the spleen and tumor microenvironment, supporting the potential of this approach for targeted cancer therapy [93].

Although the combination of TLR7/8 agonists with chemotherapeutic agents has yielded significantly enhanced therapeutic outcomes, further optimization of dose–response parameters is essential for successful clinical translation. Establishing appropriate experimental conditions to accurately assess drug retention time and deep tissue penetration within tumors is critical. These insights will help define precise pharmacokinetic and pharmacodynamic (PK/PD) profiles, thereby improving therapeutic efficacy. Additionally, conducting preclinical studies using patient-derived xenograft (PDX) tumor models in humanized mice may strengthen the translational potential of these combination strategies for clinical applications.

### 5.3. Combination with Phototherapy

Phototherapy, encompassing photothermal therapy (PTT) and photodynamic therapy (PDT), is a widely used non-invasive strategy for ablating primary tumor masses. Despite its promise, phototherapy alone often fails to achieve complete tumor eradication, necessitating combination approaches to enhance therapeutic efficacy.

Recent studies have explored the integration of phototherapy with immunostimulatory agents and inhibitors to boost antitumor responses. For example, virus-like nanoparticles (VLPs) derived from bacteria, yeast, plants, and mammalian cells-known for their biocompatibility and scalability-have been investigated as delivery vehicles for cancer therapy [94]. In a proof-of-concept study, Christian Isalomboto Nkanga et al. utilized tobacco mosaic virus (TMV) particles, chemically modified with polydopamine (PDA) for photothermal conversion, and loaded with the TLR7 agonist 1V209. This formulation significantly improved survival in C57BL/6 mice with B16F10 dermal melanoma (60% vs. 20% in controls) and enhanced systemic antitumor immunity via increased tumor-specific T cells [95]. Yosothamani et al. developed polyaniline (PANi)-based nanoparticles targeting estrogen receptor-positive (ER+) breast cancer cells. These nanoparticles were functionalized with endoxifen (END) and loaded with the TLR7 agonist R837. Combined with anti-PD-L1 therapy and NIR laser irradiation, the treatment elevated CD8^+^ and CD4^+^ T cells, with the most pronounced immune activation observed in the group receiving all three modalities [96].

Qin et al. fabricated an NIR-activated hydrogel using agarose and the photothermal agent M-4, co-delivering R837 and doxorubicin (DOX). This synergistic approach enhanced DC maturation and activated T cells in both primary and secondary tumors, demonstrating robust systemic immune responses and reduced tumor burden [97]. Mei and colleagues employed naturally occurring polymers such as alginate and collagen to create self-assembling hydrogels encapsulating R837 and methylene blue (MB). These hydrogels exhibited shear-thinning and self-healing properties, enabling sustained drug release and localization, and showed promising photothermal-induced antitumor immune responses [98].

Yue et al. designed a core–shell nanoplatform composed of zinc porphyrin and mesoporous nanoparticles (MPSNs@R837), integrating R837 and anti-PD-L1 for enhanced photoimmunotherapy. The combination of PTT, PDT, and immunotherapy significantly inhibited tumor growth and promoted DC maturation and T cell activation, outperforming monotherapies [99]. Jiang et al. developed a photoactivable polymeric nanoagonist (APNA) using an NIR-II absorbing semiconducting polymer functionalized with R848 via a thermos-responsive linker. Under NIR-II irradiation, APNA induced potent cell death and activated DCs, leading to elevated CD8^+^ and CD4^+^ T cells in distant tumors and metastatic sites, including the lungs and liver. This strategy demonstrated strong potential for nanovaccine development [100].

**Figure 6:**
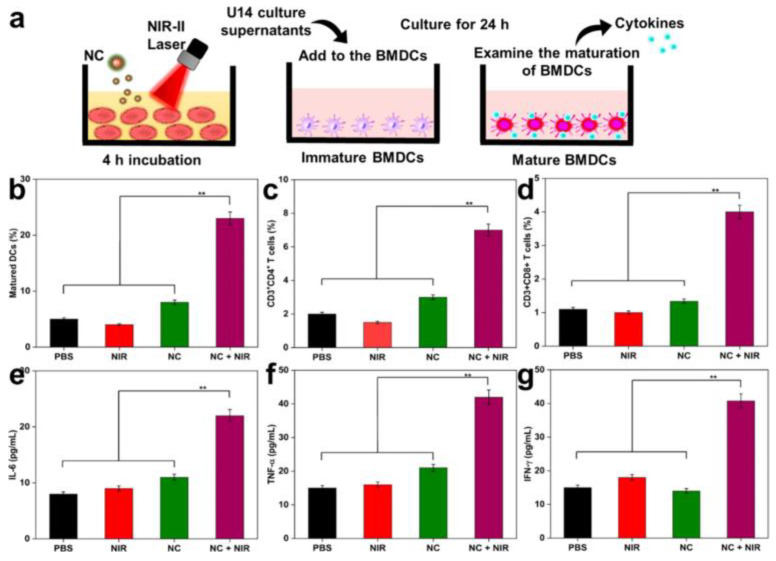
(**a**) In vitro approach to mature BMDC. (**b**) Maturation of DCs in tumor-draining lymph nodes with various treatments. (**c**,**d**) Quantification of CD4^+^ T-cells and CD8^+^ T-cells (gated on CD3^+^) in tumors after 10 days of various treatments (analyzed by Flow Cytometry). (**e**–**g**) ELISA analysis of IL-6, TNF-α, and IFN-γ of mice after various treatments. Laser treatment was 1064 nm of 1 W/cm^2^ for 5 min. The statistical significance of the experimental data was assessed via the mean ± SE and Student’s *t*-test. Data are presented as the mean ± SD (*n* = 5); and ** *p* < 0.01. Adapted with permission from [101]. Copyright © 2023, American Chemical Society. NIR-II: second Near-Infrared, BMDCs: bone marrow-derived dendritic cells.

Further advancing this concept, another group synthesized NIR-II responsive nanocomposites (NCs) from polypyrrole, functionalized with TAT peptides and hyaluronic acid (HA) for nuclear targeting and R848 delivery. These NCs exhibited excellent photothermal stability and induced nucleus-specific cell death under NIR-II irradiation. Immune activation markers such as calreticulin and HMGB1 were upregulated, alongside increased DC maturation and pro-inflammatory cytokines (IL-6, TNF-α, IFN-γ), confirming the efficacy of this nanovaccine platform (Figure 6) [101].

In another study, PANi nanoparticles were coated with poly(vinylpyrrolidone) (PVP) to prevent aggregation and modified with RGD peptides for targeted delivery to cancer cells. R837 was encapsulated to enhance antitumor immune responses. Flow cytometry analysis revealed significantly higher populations of CD4^+^ and CD8^+^ T cells compared to other treatment groups. This synergistic strategy also promoted DC activation and reduced tumor recurrence relative to NIR alone and untreated controls, suggesting its potential as an effective nanovaccine platform [102].

**Figure 7:**
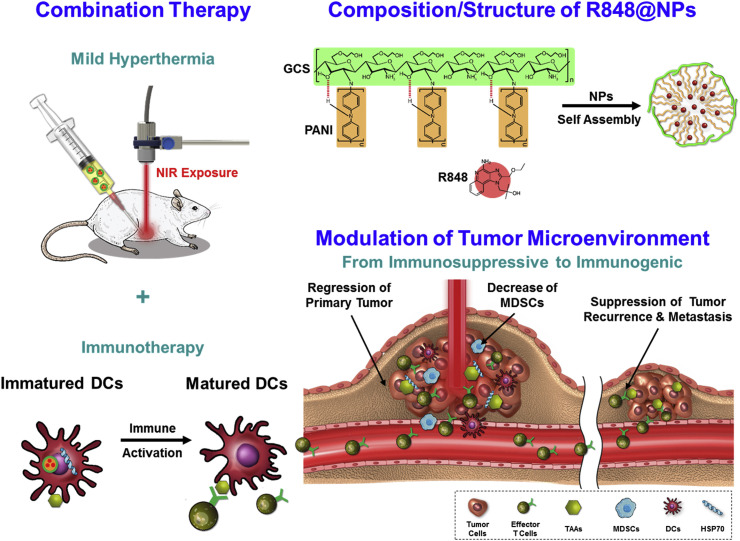
Schematic diagram of treatment strategy and mechanisms of immune responses and fabrication of self-assembled nanoparticles of PANI-GCS loaded with R848. Reprinted with permission from [103]. Copyright © 2019, Elsevier Ltd. PANI: polyaniline, GCS: glycol-chitosan, R848: an immune adjuvant resiquimod.

Chen et al. proposed that low-temperature PTT may be more effective and safer than high-temperature PTT, which can damage surrounding healthy tissues and immune cells. To address the limitations of deep tissue penetration and heat generation, they designed R848-loaded PANi-conjugated glycol-chitosan nanoparticles (R848@NPs). These acted as in situ low-temperature cancer vaccines. The treatment significantly upregulated CD80 and CD86 expression on bone marrow-derived DCs, and increased IL-6 and TNF-α levels, indicating effective immune activation. In vivo studies using Balb/c mice exposed to NIR laser at 0.9 W/cm^2^ and 2.0 W/cm^2^ showed that low-temperature treatment with R848@NPs most effectively inhibited tumor growth and enhanced effector T cell responses. This strategy elicited systemic antitumor immunity and holds promise for clinical translation (Figure 7) [103].

Moreover, Meng and colleagues demonstrated that mild hyperthermia therapy could significantly inhibit tumor growth, metastasis, and enhance antitumor immune responses in melanoma. They fabricated a thermoresponsive hydrogel system (R837@PDA@CGP) by embedding R837 nanocrystals coated with PDA into chitosan hydrogel. Nanocrystals were chosen for their superior drug loading capacity [104]. In vivo studies using B16F10 melanoma models in C57BL/6 mice showed that a single dose of R837@PDA@CGP followed by NIR irradiation led to significant tumor reduction and enhanced DC maturation. CD8^+^ T cell populations increased to 15.90 ± 2.88%, and IFN-γ levels were elevated, confirming the formulation’s ability to boost antitumor immunity [105].

**Figure 8:**
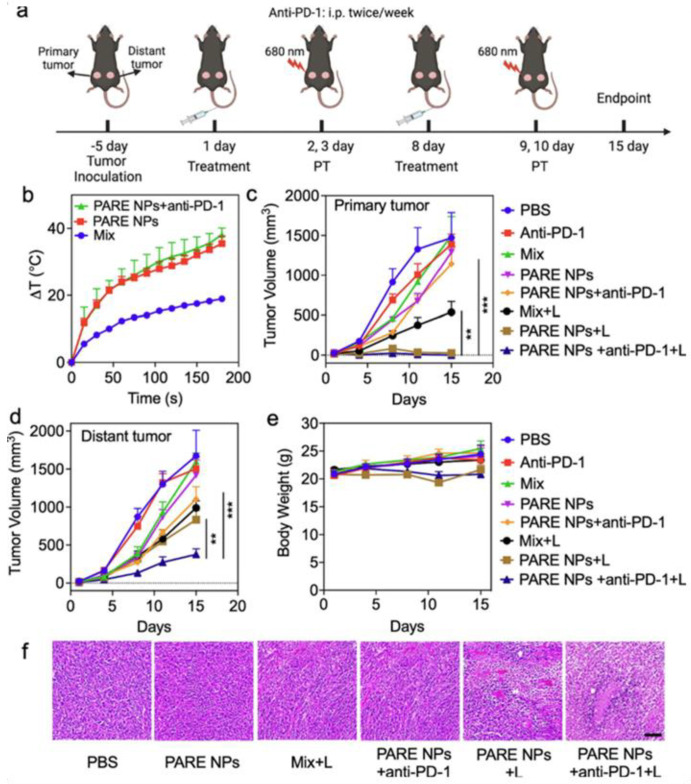
Evaluation of antitumor efficacies of developed NPs in in vivo studies. (**a**) The illustration of tumor establishment in HNSCC tumor mouse model and the schedule for subsequent treatments with both laser and therapeutics. The laser was treated with 0.5 W/cm^2^ for 3 min. (**b**) The photothermal ablation effects of the tumor after the treatments of Mix, PARE NPs alone, and triple combination. (**c**,**d**) The changes in tumor volume of both primary tumors and the distal tumor. (**e**) Assessment of Body weight changes in tumor-bearing mice. (**f**) Assessment of H&E staining of the tumor tissue after various treatments. The scale bar is 100 μm. Statistical analysis was performed by Student’s *t*-test for two groups, and one-way ANOVA for multiple groups. Adapted with permission from [106]. Copyright © 2023, Elsevier B.V. PARE NPs: Self-assembled nanoparticles of prodrug derived from pyropheophorbide-A, PA, a photosensitizer, and R848, an immune adjuvant resiquimod.

Qu et al. developed prodrug-based nanoparticles (PARE NPs) by conjugating pyropheophorbide-A (PA) and R848. These self-assembling NPs generated ROS and photothermal effects, enhancing immune responses. In SCC-7 tumor models, laser treatment combined with anti-PD-1 therapy significantly reduced both primary and distant tumors. CD4^+^ T cell populations increased to >60%, and DC maturation in lymph nodes was markedly improved, demonstrating systemic immunity and potential for treating head and neck cancers (Figure 8) [106].

Wang et al. reported acidic-responsive polymeric nanoparticles (ARNPs) engineered with R848 and PA to activate in tumor microenvironments. Under 671 nm laser irradiation, ARNPs produced ROS, triggered immunogenic cell death (ICD), and released R848 to enhance antigen presentation. In CT-26 colorectal tumor models, ARNPs combined with laser and checkpoint blockade therapy led to tumor regression and extended survival (50% on day 32). Increased CTL infiltration and reduced Tregs confirmed the efficacy of this combinatory approach [107].

Another promising strategy involved a liposomal formulation (Lipo@IR808@Loxo) combining NIR-responsive IR808 and loxoribine. This system enhanced PTT efficacy and antigen release under NIR irradiation, while loxoribine activated APCs. In 4T1 tumor-bearing mice, Lipo@IR808@Loxo + Laser treatment completely eradicated tumors and induced CRT expression, confirming ICD. Anti-PD-1 therapy further boosted immune responses, leading to the elimination of bilateral tumors [108].

Revuri et al. introduced BAGEL-R848, an injectable thermosensitive hydrogel composed of MnO_2_ NPs, R848, and hyaluronic acid/pluronic F127. Designed for localized delivery, BAGEL-R848 showed high photothermal conversion efficiency and significantly reduced both primary and distant tumors in 4T1 models. Elevated levels of TNF-α, IL-6, IFN-γ, and IL-12p70 confirmed strong immune activation, highlighting its potential as a cancer treatment platform [109].

An extensive study reported that PDT alone is insufficient for complete cancer treatment. Researchers demonstrated that combining PDT with immunostimulatory agents-such as poly(I:C), R848, and macrophage inflammatory protein 3 (MIP3), enhanced antitumor immunity while minimizing systemic side effects. PLGA nanoparticles were used to deliver these agents locally. In MC38, CT26, and TC-1 tumor models, combination therapy significantly delayed tumor growth compared to monotherapies. Splenocytes from treated mice showed elevated CD8^+^ T cells producing IFN-γ and TNF-α. In TC-1 models, HPV-E7-specific CD8^+^ T cells were significantly increased, confirming robust systemic immunity [110].

**Figure 9:**
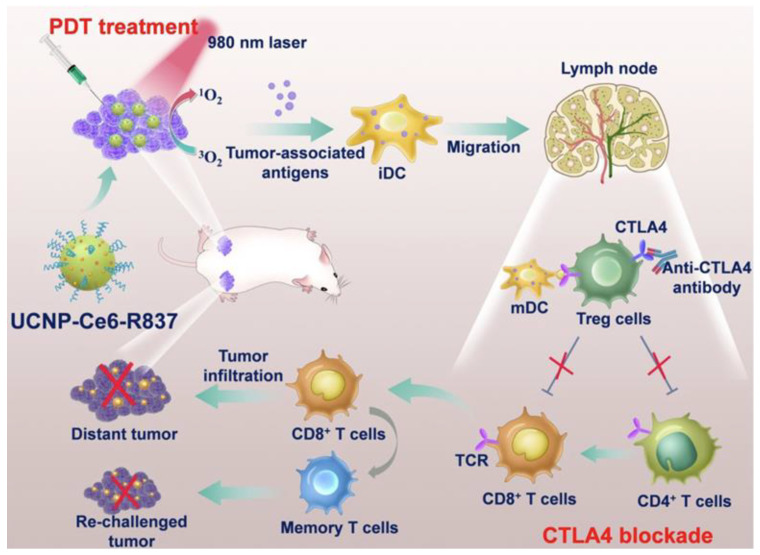
Schematic illustration of the mechanisms of the combination of NIR-mediated PDT with TLR7 agonist and checkpoint inhibitor for cancer immunotherapy. Under the NIR laser treatment, UCNP-Ce6-R837 nanoparticles initiate tumor destruction. In combination with adjuvant and checkpoint inhibitors, tumor-associated antigens promoted strong antitumor immune responses to eliminate both primary and distal tumors as well as prevent tumor recurrence. Adapted with permission from [111]. Copyright © 2017, American Chemical Society. PDT: photodynamic therapy, iDC: immature dendritic cell, iDC: mature dendritic cell, UCNP: upconversion nanoparticles, Ce6: Photosensitizer, CTLA4: critical inhibitory receptor on T cells, R837: immune adjuvant IMQ.

Xu et al. developed upconversion nanoparticles (UCNPs) with deep tissue penetration capabilities, co-encapsulating R837 and chlorin e6 (Ce6) into PEG-coated UCNPs (UCNP-Ce6-R837). This system enhanced DC maturation and cytokine production (TNF-α, IL-12), bridging innate and adaptive immunity. When combined with CTLA-4 checkpoint blockade, UCNP-Ce6-R837 treatment effectively eliminated tumors and prevented distant tumor growth, as shown in Figure 9 [111].

To address limitations in topical drug penetration for skin cancer, PDT was combined with IMQ cream in treating cutaneous squamous cell carcinoma (cSCC). Using 5-aminolevulinic acid (ALA) as a photosensitizer, patients with invasive cSCC on the lips and feet received PDT at two-week intervals, alongside daily application of 5% IMQ cream. This regimen led to complete lesion removal after several treatment cycles [112]. Collectively, these studies underscore the potential of combining phototherapy with immunomodulatory strategies to achieve synergistic antitumor effects, offering promising avenues for translational cancer immunotherapy.

These studies utilized a range of organic and inorganic photosensitizers to enhance therapeutic efficacy. Careful selection of these agents is essential to minimize the risk of adverse toxicity. Additionally, experimental designs should aim to reduce collateral damage to surrounding healthy tissues, thereby improving the safety profile of photosensitizer-based treatments.

### 5.4. Combination with Radiation Therapy and Sonodynamic Therapy

Radiation therapy has long been a cornerstone of conventional cancer treatment, with extensive clinical applications [113,114]. However, its use is often limited by adverse effects on healthy tissues, particularly when radioisotopes are administered systemically. To mitigate these side effects, radiation therapy is increasingly applied in a localized manner. For example, Dewan et al. investigated the topical application of IMQ in combination with radiation therapy (RT) in a TSA mouse model. The treatment significantly reduced tumor growth and increased infiltration of CD11c^+^ DCs, CD4^+^ T cells, and CD8^+^ T cells. The addition of RT further enhanced antitumor responses compared to IMQ alone. Moreover, pre-treatment with low-dose cyclophosphamide amplified the therapeutic effect and reduced tumor recurrence, suggesting a promising strategy for breast cancer metastasis [115]. Building on this, Adlard et al. explored systemic administration of the TLR7 agonist DSR-6434 in combination with ionizing radiation for non-dermatological tumors. In CT26 and KHT tumor-bearing mice, this approach achieved 55% complete tumor regression and improved survival. Lung metastases were significantly reduced compared to radiation alone, and CD8^+^ T cell populations were notably elevated, demonstrating enhanced antitumor immunity [116]. Cho et al. further demonstrated synergistic antitumor and anti-metastatic effects in melanoma using IMQ and ionizing radiation. The combination induced cell death via autophagy and increased autophagosome formation. Incorporating 3-methyladenine (3-MA) into the treatment regimen enhanced CD4^+^ and CD8^+^ T cell populations while reducing regulatory T cells (Tregs) and myeloid-derived suppressor cells (MDSCs), indicating potential for treating radio-resistant melanoma [117]. Dovedi et al. showed that systemic administration of R848 in combination with RT significantly improved therapeutic outcomes. Weekly intravenous doses of R848 enhanced CD8^+^ T cell-mediated responses and led to complete tumor rejection in 100% of mice rechallenged with EG7 or EL4 cells. This strategy demonstrated strong potential for treating B and T cell malignancies [118]. Ye et al. applied a similar approach in a pancreatic ductal adenocarcinoma (PDAC) model, combining R848 with stereotactic body radiotherapy (SBRT). While SBRT alone was insufficient to generate tumor antigens, the combination treatment activated CD8^+^ T cells and modulated cytokine profiles-decreasing IL-4, IL-6, and IL-10, while increasing IFN-γ, granzyme B, and CCL5. In a hepatic metastasis model, similar immunomodulatory effects were observed, supporting the potential of this strategy for metastatic PDAC [28]. Ota et al. investigated DSP-0509, another TLR7 agonist, in combination with RT in CT26 tumor-bearing mice. Systemic delivery of DSP-0509 with RT led to T cell-dependent immune activation and complete tumor elimination in 30% of treated mice—an outcome not achieved with cisplatin and RT. This approach may be particularly beneficial for tumors with poor immunogenicity or resistance to checkpoint inhibitors, warranting further clinical evaluation [119].

Sonodynamic therapy (SDT) offers a promising alternative to light-based treatments, particularly for deep-seated or inaccessible tumors [120,121]. SDT is non-invasive, safe, and capable of precise spatial targeting due to its superior tissue penetration [122,123]. Unlike phototherapy, SDT can trigger immunogenic cell death (ICD), releasing tumor-associated antigens (TAAs), inducing calreticulin (CRT) surface expression, and HMGB1 [124]. However, SDT alone is often insufficient to elicit robust immune responses [125], necessitating combination strategies. Yue et al. demonstrated that combining SDT with checkpoint blockade immunotherapy significantly enhances antitumor efficacy. They developed nanosensitizers composed of liposomes loaded with hematoporphyrin monomethyl ether (HMME) and R837 (HMME/R837@Lip). Compared to HMME@Lip alone, the combination promoted greater DC maturation and elevated cytokine levels (TNF-α, IL-6). When combined with anti-PD-L1 antibodies, this strategy suppressed both primary and distant tumors in 4T1 and CT26 models and prevented lung metastasis, providing a compelling proof-of-concept for integrating immunotherapy with non-invasive tumor treatments [126].

**Figure 10:**
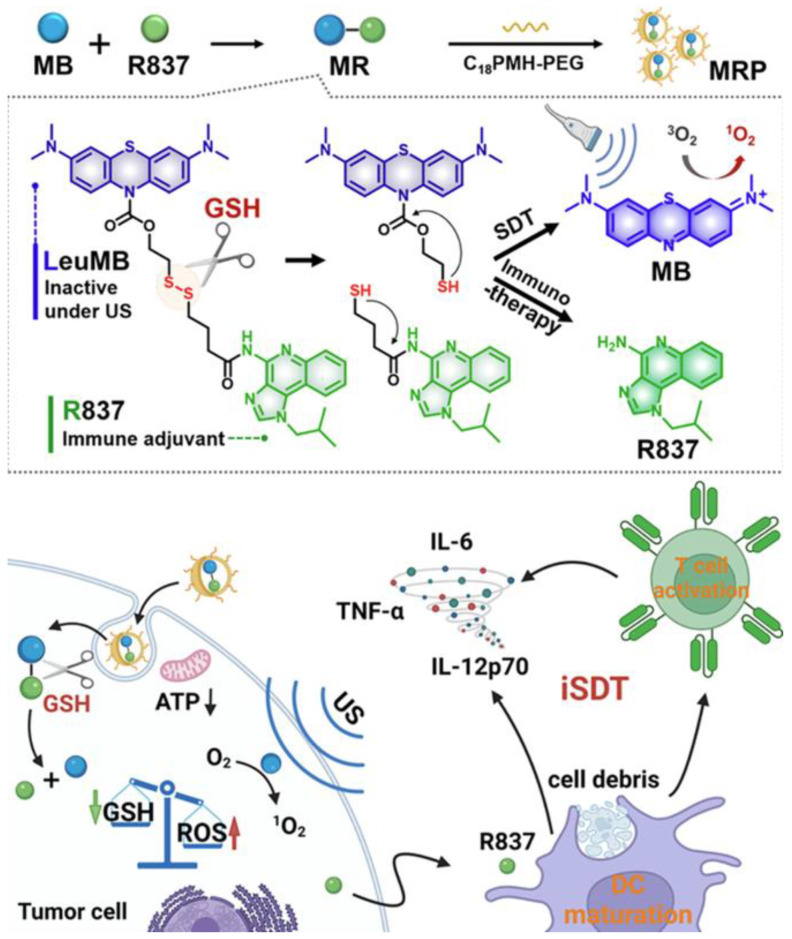
Fabrication of MB-R837-PEG (MRP) nanoparticles for glutathione-mediated immunosonodynamic treatment (iSDT). Adapted with permission from [131]. Copyright © 2022, American Chemical Society. GSH: glutathione, ATP: adenosine triphosphate, ROS: reactive oxygen species, US: ultrasound, MB: methylene blue, R837: immune adjuvant IMQ.

Recent investigations have focused on tumor-specific conditions within the tumor microenvironment (TME) to guide treatment design [127,128]. Prodrug-based strategies have garnered increasing attention due to their tumor-selective activation and reduced off-target toxicity [129,130]. For example, Lei and colleagues developed a glutathione-responsive prodrug composed of a reduced form of methylene blue and the TLR7 agonist R837. These components were encapsulated within amphiphilic polymers (C18PMH-PEG) to create drug-loaded nanoparticles, referred to as MRP. The MRP system exhibited selective activation and potent therapeutic effects at tumor sites, leading to significantly reduced tumor growth compared to individual treatments. To further enhance efficacy, anti-PD-L1 antibodies were incorporated, resulting in improved immune memory and reduced tumor recurrence (Figure 10) [131].

To overcome limitations associated with anti-PD-1/PD-L1 antibodies-such as low tumor accumulation and immunogenicity, another group developed nanoparticles via self-assembly containing Ce6, R848, and the PD-L1 inhibitor JQ1 (termed CRJ NPs). CRJ NPs combined with SDT significantly inhibited tumor growth, whereas monotherapies showed minimal effect. Cytokine analysis confirmed elevated levels of antitumor immune mediators, supporting the therapeutic potential of this strategy [132].

Although the combination of TLR7/8 agonists with sonodynamic therapy (SDT) has shown notable therapeutic enhancement, further studies are necessary to assess potential damage to healthy tissues. Particular attention should be given to evaluating whether the generation of reactive oxygen and nitrogen species (ROS/RNS) contributes to off-target toxicity during treatment. Conversely, radiation therapy remains a widely used and effective modality for solid tumors; however, its clinical application is often limited by off-target adverse effects. Incorporating radioisotopes into hydrogel-based delivery systems presents a promising strategy to minimize collateral damage to healthy tissues and improve the safety profile of radiotherapy.

Additionally, we summarize the combination of TLR7/8 agonists with other treatment modalities in Table 1.

## 6. Development of TLR7/8 Agonist-Based Cancer Vaccines and Their Clinical Translation

**Figure 11:**
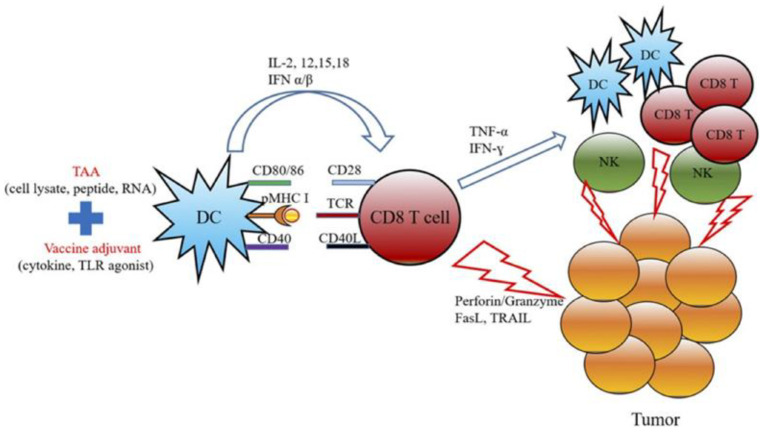
The schematic diagram represents the mechanism of actions for cancer vaccine development by combining the treatment of TAAs and cancer adjuvants. Adapted with permission from [138]. Copyright © 2019, Elsevier.

Cancer vaccine development typically involves combining tumor-associated antigens (TAAs) with adjuvants to stimulate cytotoxic CD8^+^ T cells capable of targeting cancer cells. These components initiate DC maturation, which subsequently enhances tumor-specific T cell responses [136]. Mature DCs express co-stimulatory molecules and release pro-inflammatory cytokines, presenting TAAs to activate cytotoxic T cells and NK cells [137]. This cascade further amplifies immune activation across DCs, T cells, and NK cells. A mechanistic illustration of cancer vaccine pathways is presented here (Figure 11) [138].

Although autologous tumor cell-based vaccines have shown efficacy, their clinical application is limited by the need for patient-specific tumor tissue. As an alternative, peptide- or protein-based vaccines derived from tumor cells have been extensively explored. However, these approaches have generally failed to elicit robust T cell responses [139], highlighting the need for additional immune stimulants. Among these, Toll-like receptor (TLR) agonists have been widely investigated as adjuvants to enhance cancer vaccine efficacy. TLR7/8 agonists, in particular, can directly activate the innate immune system, promote antigen presentation by DCs, and stimulate strong T cell responses [140]. Despite their promise, FDA-approved cancer vaccines incorporating TLR7/8 agonists remain limited.

To address this gap, Geoffrey et al. proposed polymer-based particulates functionalized with TLR7/8 agonists to improve vaccine efficacy. Their study demonstrated enhanced pharmacokinetic profiles and prolonged accumulation in draining lymph nodes (dLNs), along with broad antibody responses and robust T cell activation [141]. In another study, Shubhmita et al. employed a multi-adjuvant strategy combining ovalbumin with DMXAA and compound 522, which significantly enhanced CD8^+^ T cell and NK cell responses, leading to tumor suppression and improved survival compared to controls [75]. R848-loaded nanoemulsions combined with anti-PD-1 antibodies have also shown synergistic antitumor effects. This formulation promoted DC maturation and T cell proliferation, highlighting its potential as a cancer vaccine platform [142]. Kim et al. developed a sustained-release liposomal formulation of R848, chemically conjugated with cholesterol and cationic lipids. Compared to the commercial adjuvant AS01, this nanovaccine demonstrated superior antitumor efficacy and tumor inhibition [143].

Due to their immunomodulatory properties, small-molecule TLR7/8 agonists have progressed into clinical evaluation following promising preclinical results. For example, SHR2150 (TLR7 agonist) was combined with chemotherapy and PD-1 or CD47 antibodies in patients with metastatic solid tumors (NCT04588324). RO7119929 was orally administered to assess safety, PK/PD, and antitumor activity in patients with hepatic metastases (NCT04338685), involving 55 participants in a Phase I study. IMQ was evaluated for breast and skin metastases via topical application over eight weeks (NCT00899574), with 10 patients enrolled. The treatment was well tolerated, and two patients showed localized cytokine production [144]. IQ was also combined with local radiation therapy and cyclophosphamide (CTX) to assess systemic responses in skin metastases (NCT01421017), involving 31 patients in a Phase I/II trial. The study demonstrated safety and systemic antitumor immunity [145]. IQ combined with laser therapy was tested in patients with stage III or IV melanoma and skin metastases (NCT00453050), enrolling 70 patients. 852A, administered subcutaneously, showed antitumor effects in breast, ovarian, and cervical cancer patients (NCT00319748) [146]. One patient completed 24 doses with an additional 17 doses, and the study reported sustained tolerance and moderate antitumor activity. Another report indicated that two-thirds of patients with resistant metastatic melanoma treated with 852A experienced disease stabilization without further tumor growth or significant side effects [147].

Additional clinical trials include DSP-0509, tested in patients with advanced solid tumors either alone or with pembrolizumab (NCT03416335), enrolling 36 patients. BNT411, evaluated in patients with extensive-stage small cell lung cancer (ES-SCLC) as monotherapy or in combination with atezolizumab, carboplatin, and etoposide (NCT04101357). No severe adverse events or dose-limiting toxicities were observed, and one patient responded positively among the 11 enrolled. DN1508052-01, administered subcutaneously in patients with solid tumors (NCT03934359), involved 19 participants. The study assessed safety, maximum tolerated dose, and PK, revealing a favorable PK profile lasting up to two months.

VTX-2337, a TLR8 agonist, has been evaluated in multiple trials, such as Trials for B-cell lymphomas were terminated (NCT01289210, NCT02650635), but studies in HNSCC (NCT03906526, NCT01334177, NCT01836029), ovarian cancer (NCT01666444, NCT01294293, NCT02431559), and advanced solid tumors (NCT02650635) were completed. Combination with cetuximab showed no dose-limiting toxicities and led to increased plasma cytokine levels and NK cell activation (NCT01836029) [148]. Gregory et al. reported elevated biomarkers (IL-6, G-CSF, MCP-1, MIP1-β) in late-stage cancer patients and healthy volunteers, consistent with preclinical PK/PD profiles [149]. A trial combining VTX-2337 with nivolumab in patients with SCCHN is currently recruiting (NCT03906526).

MEDI9197, another TLR7/8 agonist, was tested in patients with advanced solid tumors. It was combined with durvalumab or palliative radiation therapy to evaluate antitumor activity. The study showed increased intratumoral CD8^+^ and PD-L1^+^ cells following treatment (NCT02556463) [150].

In another study, three DC vaccine formulations—tumor lysate + DCs, tumor lysate + DCs + 0.2% resiquimod, and tumor lysate + DCs + polyICLC—were evaluated in patients with glioma to determine the most effective approach (NCT01204684). This Phase II trial enrolled 24 patients.

Topical application of resiquimod gel at two concentrations was tested in patients with cutaneous T cell lymphomas to assess safety and antitumor activity (NCT01676831). Thirteen patients participated in Phase I and II trials. Two additional trials (NCT00470379, NCT01748747) evaluated the efficacy of peptide vaccines combined with resiquimod in patients with stage II–IV melanoma following surgical tumor removal.

BM201, administered intratumorally in 17 patients with refractory or metastatic solid tumors (NCT06368960), demonstrated a sustained plasma PK profile and significant tumor reduction in most patients. Notably, 29.4% of subjects exhibited potent abscopal effects. The study showed a favorable safety profile and recommended further evaluation of dose-dependent toxicity.

BDB001 was administered intravenously as monotherapy or in combination with pembrolizumab (NCT03486301) or atezolizumab (NCT04196530) in Phase I trials. The primary objective was to assess safety and tolerability, while secondary endpoints included efficacy and PK/PD. Approximately 29% of subjects receiving dose levels 3 and 4 showed sustained and prominent clinical responses. The treatment was well tolerated and supported robust systemic immune activation, prompting recommendations for dose escalation trials [151]. An ongoing trial is evaluating BDB001 in combination with atezolizumab and radiation therapy in patients with solid tumors (NCT03915678). A related compound, BDB018, is being tested as monotherapy or in combination with atezolizumab in a Phase I trial for solid tumors (NCT04840394).

BDC-1001, another TLR7/8 agonist, is under investigation as a single agent or in combination with nivolumab for HER2-expressing advanced malignancies (NCT04278144). This Phase I/II trial enrolled 75 patients and includes four parts: dose determination (Part 1), dose escalation with nivolumab (Part 2), efficacy evaluation of monotherapy (Part 3), and combination efficacy assessment (Part 4).

LHC165 is being evaluated as monotherapy or in combination with PDR001 for advanced malignancies (NCT03301896). This trial is active but not currently recruiting.

Finally, NKTR-262, administered intratumorally in combination with nivolumab, was tested in patients with solid and metastatic tumors. The study was terminated after Phase I due to results (NCT03138733).

Together, these studies highlight the translational potential of TLR7/8 agonists in cancer vaccine development. Continued innovation in formulation and delivery strategies will be critical to overcoming current limitations and advancing these platforms toward broader clinical use. We also summarize ongoing and completed trials involving TLR7/8 agonists in Table 2 [22,49]. Due to the limited publicly available clinical data for many of these studies, we have strived to include patient participation, cancer-specific treatments, trial status, and study outcomes where available.

## 7. Conclusions and Future Directions

TLR7/8 agonists have emerged as promising candidates in cancer immunotherapy due to their ability to activate innate immunity and stimulate robust antitumor responses. These agonists have been widely explored not only for cancer treatment but also for combating infectious diseases. Synthetic small-molecule TLR ligands have demonstrated potent immunostimulatory effects in both preclinical and clinical settings, and their role as vaccine adjuvants continues to gain traction.

Despite encouraging results, the limited therapeutic efficacy of TLR7/8 agonists has prompted deeper investigation into their molecular mechanisms of action. One major challenge is systemic toxicity, which can hinder clinical outcomes. Advances in structural biology and structure–activity relationship (SAR) analyses have helped elucidate the molecular interactions of these agonists, paving the way for more targeted and safer designs.

However, challenges remain. Optimal dosing schedules and timing intervals are critical to avoid adverse effects, including the risk of autoimmunity due to repeated dosing and TLR tolerance. Extensive research is ongoing to fine-tune these parameters and ensure sustained pro-inflammatory immune activation [64,152,153,154]. An emerging area of interest in immunotherapy research is the impact of circadian biology on Toll-like receptor (TLR) expression and agonist efficacy. Circadian rhythm-dependent regulation of TLR expression and activation presents a significant challenge, as TLR-mediated immune responses fluctuate throughout the 24 h cycle. These temporal variations in receptor availability and signaling efficiency can influence cytokine production and antigen presentation, resulting in inconsistent immunological outcomes. Such variability undermines experimental reproducibility and complicates the optimization of dosing schedules for TLR-based vaccines and immunotherapies. Consequently, a deeper understanding of circadian influences on TLR signaling is essential for achieving reliable and effective immune activation [155,156].

Further investigation is warranted to elucidate the mechanisms underlying the development of innate immune-targeted therapies. Pathways such as autophagy and inflammasome activation may influence pro-tumor or antitumor responses depending on cancer type, tumor microenvironment, and disease stage. Consequently, targeting macrophages or inflammasome components presents a promising anticancer strategy [157,158]. Numerous studies have explored the blockade of inhibitory immune checkpoints, including anti-CTLA-4 and anti-PD-1/PD-L1 antibodies, which can alleviate immunosuppressive effects. However, these therapies have shown significant clinical responses only in a limited subset of patients [159]. In this context, TLR7/8 agonists—either as monotherapies or in combination with other immunostimulatory agents—have demonstrated the ability to suppress tumor growth and enhance immune activation. Notably, combining TLR agonists with immune checkpoint inhibitors such as PD-1/PD-L1, CTLA-4, TIGIT, and LAG3 has yielded synergistic effects and improved therapeutic outcomes across various cancer models [160,161,162].

In addition to combining multiple immunostimulatory agents and multiple treatment modalities, the development of optimized formulation strategies is essential to minimize systemic toxicity. The design of advanced delivery systems plays a critical role in achieving sustained, targeted, and curative cancer vaccine formulations. Continued progress in the development of TLR7/8 agonists—either as monotherapies or in combination with other therapeutic approaches—holds promise for improving treatment outcomes and enhancing survival rates across diverse patient populations.

## Figures and Tables

**Table 1:** Summarization of combining TLR 7/8 agonists with other treatment modalities.

Treatment Strategies	TLR 7/8 Agonists	Delivery Systems/ Delivery Routes	Cancer Treatments	Study Outcomes/Safety Findings	Ref.
Immunomodulators	522	Nanoparticles Subcutaneously oral gavage Intraperitoneally (IP)	MB49 and B16-OVA murine tumor models	• Resulted in the depletion of MDSCs and Tregs • Reduction in PD-L1^high^ M2 macrophages • Enhanced the CD44^high^ CD8+ T cells	[67]
	R848	Nanoemulsion (NE) Intratumorally (i.t)	B16F10-OVA tumor-bearing mice TC-1 cervical tumor model	• Significant reduction in the tumor • Reprogrammed tumor microenvironment • Confirmed the abundant recruitment of T cells in tumor sites • Notably inhibited tumor rechallenged compared to single or other treatments • No systemic toxicity	[68]
	IMD	nMOF i.t	Colorectal tumor model (CT26 cells)	• Efficiently repolarized to M1 macrophages • Altered the immunosuppressive tumor microenvironment • No systemic toxicity	[69]
	Analog of R848	Conjugated nanoparticles IP	Murine colon adenocarcinoma model	• Murine colon adenocarcinoma model • Significantly reduced tumor growth • Extended survival rate • Minimized the systemic toxicity of free drugs	[71]
	R848	Implant	Colorectal Cancer	• Eradication of tumors after surgery for up to 150 days • Inhibited distal tumor growth with the production of immune memory responses. • Revealed improved NK cells infiltration, DCs maturation, and T cells infiltration in the first few days • No obvious toxicity	[72]
	IMD	Conjugated nanoparticles peritumoral (IT)	4T1 (mouse breast cancer) tumor B16 melanoma mouse model	• Significant reduction in tumor growth • Dramatically reduced systemic toxicity • Potentially activated DCs in tumor LNs • Notably increased CD8+ T cells in tumor LNs and spleen • No significant systemic toxicity	[133]
	R848	Liposomes IP	Ovarian tumor-bearing mice (ID8 cells)	• Specifically targets TAMs and activated macrophages • Efficiently repolarized to M1 macrophages. • Showed complete tumor rejection till 250 days	[73]
	R848	Mesoporous nanoparticles (MSN) Intravenous (i.v)	CT26 colon carcinoma, B16 melanoma	• Systematically release of αPD-L1, HMGN1 and R848 • Averted immunosuppression and T cell exhaustion • Complete tumor elimination in colon tumors • No apparent toxicity	[74]
	522	Subcutaneous	Melanoma and bladder tumor models (B16F10, MB49)	• Reducing tumor growth with increasing survival rate • Produced higher antigen-specific CD8+ T cell and NK cell responses	[75]
Chemotherapy	R848	Liposomes IP	CT26 murine colorectal tumors	• Significantly augmented infiltration of CD+ T cells • Significantly inhibited the growth of the tumor • No significant toxicity	[89]
	R848	Micelles IP	Gastric Cancer MFC tumor-bearing 615 mice	• Systematically released of R848 at specific sites • Typically, repolarized M2 macrophages to M1 • Increased the population of CD4+ and CD8+ cells • No significant toxicity	[90]
	R837	Hydrogels Nanoparticles i.t	4T1 bearing tumor model	• Remarkably increased the proportions of (CD8+ CTL) • Significant tumor inhibition effect • Low toxicity to both normal organs and systemic	[91]
	IQ	Hydrogel Nanoparticles i.t	Breast cancer (4T1 cells)	• Eliminated the tumor after 3 months • Memory T cell proliferation and DC maturation were significantly elevated • Survival rate increased compared to the untreated	[92]
	R837	Hydrogels subcutaneous	Melanoma (B16F10 cells)	• Effectively suppressed the melanoma growth • The levels of IFN-γ and TNF-α were high in the spleen and the tumor area	[93]
Phototherapy	1V209	TMV particles i.t	B16F10 dermal melanoma	• 60% of C57BL/6 mice with B16F10 dermal melanoma survived • Significant improvement in tumor-specific T cells in splenocytes	[95]
	R837	Polyaniline-based NPs i.t	MCF-7 bearing tumor model	• Suppressed tumor growth • CD8+ T and CD4+ T cells significantly elevated • No obvious toxicity	[96]
	R837	Hydrogel i.t	Breast cancer (4T1 cells)	• Showed a significant increase in DC maturation and CD4+ T, CD8+ T cells • Elicited robust anticancer immune responses	[97]
	R837	Hydrogel i.t	Breast cancer (4T1 cells)	• Inhibited both primary and distal tumor growth • Demonstrated strong immune responses • No negligible toxicity	[98]
	R837	Mesoporous nanoparticles i.v	4T1 tumor-bearing mice	• Efficiently accumulated at tumor sites • Showed a stronger antitumor effect	[99]
	R848	Polymeric nanoparticles i.v	4T1 tumor-bearing mice	• Remarkably elevated CD4+ T, CD8+ T cells in distant tumors, lungs, and liver • Dramatic reduction in tumor growth	[100]
	R848	Polypyrrole (PPy)-based NCs i.t	U14 tumor-bearing mice	• Increased DC maturation • Significantly increased IL-6, TNF-α and IFN-γ • No obvious toxicity	[101]
	R837	PANI NPs i.v	U14 tumor-bearing mice	• Enhanced CD3+ CD4+ and CD3+ CD8+ T cells • Significantly promoted dendritic cells (DCs) and remarkably inhibited tumor growth • No obvious toxicity	[102]
	R848	PANI-conjugated glycol-chitosan NPs i.t	CT26 tumor mouse model	• Upregulated the levels of IL-6 and TNF-α • Significantly inhibited tumor growth • Enhanced T-cell activation at tumor sites	[103]
	R837	PDA NPs-embedded chitosan hydrogel i.t	Melanoma (B16F10 cells)	• Significant tumor inhibition • DCs’ maturation was much higher than in other study groups • CD3+ CD8+ enhanced to 15.90 ± 2.88% • Low toxicity	[105]
	R848	Prodrug-based NPs i.v	SCC-7 tumor mouse models	• Significant reduction in both primary and distal tumor • CD4+ T cells dramatically increased • No discernible tissue damage	[106]
	R848	Polymeric nanoparticles i.v	Colorectal tumors CT26 xenograft tumor models	• Remarkably inhibited tumor growth • Higher infiltration of CTLs and lower infiltration of Tregs in the tumor • No obvious damage to organs	[107]
	Loxoribine	Liposome i.v	4T1 tumor-bearing mice	• Significant induction of CRT • Drastically hindered the tumor growth	[108]
	R848	Hydrogel i.t	4T1 tumor-bearing mice	• Combined treatment drastically reduced both primary and distant tumors • Cytokine levels were significantly increased	[109]
	poly(I:C), R848	PLGA nanoparticles i.t	MC38, CT26 or TC-1 tumors bearing mice	• Demonstrated significant delays in tumor growth • Increased population of CD8+ T cells	[110]
	R837	UCNPs i.t	CT26 tumor mice model	• Stimulated DC maturation and produced cytokines • Effectively eliminated tumors as well as produced antitumor immunity	[111]
	IMQ	Cream	Skin cancer	• Complete removal of lesions after a few cycles of treatment • Well tolerated and no adverse effect	[112]
Radiation and sonodynamic therapy	IMQ	Placebo cream	TSA mouse tumor model	• Enriched CD11c, CD4+, and CD8+ cell infiltration in tumors • Significant improvement in tumor growth inhibition and recurrence by combined treatment	[115]
	IMQ	Subcutaneous	B16F10 or B16F1 melanoma	• Presented cell death via the autophagy pathway • The populations of CD4+ T and CD8+ T cells were dramatically enriched in the combined treatment compared to a single treatment	[117]
	DSR-6434	Intravenous	CT26 or KHT tumor-bearing mice	• Delayed tumor growth compared to treatment alone • Enhanced infiltration of cytotoxic CD8+ T cells and NK cells • Increased cytokines within the tumor • Improved survival rates without significant systemic toxicity	[116]
	DSR-29133	Intravenous	Renal cancer (Renca), metastatic osteosarcoma (LM8), and colorectal cancer (CT26)	• Tumor growth was dependent on CD8+ T cells rather than CD4+ T and NK/NKT-cells • Demonstrated the impact of the dose-scheduling effect of the combination treatment	[134]
	DSP-0509	Intravenous	4T1 and CT26 bearing tumor model	• 30% of tumors in bearing mice resulted in complete tumor reduction • Significantly improved the antitumor activity	[119]
	3M-011 (854A)	IP	Colorectal (CT26 cells) and pancreatic cancer (Panc-02 cells)	• Resulted in the complete elimination of the tumors in 50% of the mice compared to alone • Tumor growth was significantly delayed by combined treatment. • Presented CD11c+ DC that mediated the therapeutic effects through the activation and priming of NK and CD8 T cells	[135]
	R848	Intravenous	Mouse models of lymphoma (EG7, EL4, or A20 cells)	• Significantly improved the therapeutic effects compared to either a single dose or control mice with a combination of RT • Combined treatment was dependent on the activity of cytotoxic CD8+ T cells • 100% of mice showed complete tumor rejection after the combination treatment of R848 (dosed weekly) and fractionated RT	[118]
	R848	Intravenous	Murine models of pancreatic cancer	• Resulted in significant activation of immune cells, including CD8+ T cells, in the pancreatic TME • Remarkably decreased in IL-4, IL-6, and IL-10 and an increased in IFN-*γ*, granzyme B, and CCL5	[28]
	R837	Liposomes i.v	4T1 or CT26 tumor-bearing mice	• Effectively prevented lung metastasis • Significantly suppressed both primary and distant tumors	[126]

**Table 2:** List of TLR 7/8 agonists applied for clinical settings.

TLR 7/8 Agonists	Route of Administration	Application Area	Key Findings/Safety Findings	Company	Phase	Trial ID
Imiquimod	Toipcal	Breast cancer	Well tolerated Cytokine production at tumor sites and tumor regression	NYU Langone Health (New York, NY, USA)	II	NCT00899574
	Toipcal	Breast cancer	Not significant clinical progress	NYU Langone Health	I/II	NCT01421017
	Topical	Melanoma	Enhancing vaccination	3 M (Tulsa, OK, USA)	I	NCT00453050
852A	SC	Breast, ovarian, endometrial, and cervical cancers	Revealed antitumor activities	Masonic Cancer Center (Minneapolis, MN, USA), University of Minnesota	II	NCT00319748
DSP-0509	i.v infusion	Advanced solid tumors	Not reported	Sumitomo Pharma Oncology, Inc. (Marlborough, MA, USA)	I/II	NCT03416335
BNT411	i.v	Solid tumors, ES-SCLC	Investigated for the evaluation of safety, tolerability, clinical benefit, pharmacokinetic (PK), and pharmacodynamic data for phase II	BioNTech Small Molecules GmbH (Planegg, Bavaria, Germany)	I	NCT04101357
DN1508052	SC	Advanced Solid Tumors	Not reported	Shanghai De Novo Pharmatech Co., Ltd. (Shanghai, China)	Ib/II	NCT03934359
VTX-2337	i.t	Low-grade B-cell lymphomas	Determining the safety and effectiveness of a combination of radiation	Celgene (Summit, NJ, USA)	I/II	NCT01289210
	SC	Ovarian epithelial, Fallopian tube, or peritoneal cavity cancer	Not reported	Celgene	I	NCT01294293
	SC	Squamous Cell Carcinomas of the Head and Neck (SCCHN)	Not reported	Celgene	I	NCT01334177
		Epithelial ovarian, Fallopian tube or primary peritoneal cancer	Did not enhance clinical outcomes with combination treatment Identified significant changes in the OS of participants based on injection site reactions	Celgene	II	NCT01666444
		Squamous cell carcinoma of the head and neck	Not reported	Celgene	II	NCT01836029
	i.v infusion	Ovarian cancer	Determined a well-tolerated safety profile and promising efficacy	Celgene	I/II	NCT02431559
	SC	Advanced solid tumors	Not reported	Celgene	Ib	NCT02650635
	SC, i.t	Head and neck cancer	Not reported	Celgene	Ib	NCT03906526
MEDI9197 (3 M-052)	i.t	Solid tumors or CTCL	Reported induced systemic and intratumoral immune activation	MedImmune LLC (Gaithersburg, MD, USA)	I	NCT02556463
Resiquimod	Topical	Melanoma vaccination	Enhanced vaccination	Mayo Clinic (Rochester, MN, USA)	I/II	NCT01748747
	Topical	Melanoma	Enhanced vaccination	Mayo Clinic	II	NCT00470379
BM201	i.t	Refractory or metastatic solid tumors	Favorable safety profile Demonstrated antitumor activity	InnoBM Pharmaceutical Co., Ltd. (Suzhou Industrial Park, Jiangsu, China)	I	NCT06368960
BDB001	i.v	Advanced solid tumors	Well-tolerated 29% response rate in anti-PD-(L)1 refractory tumors Robust immune activation	Seven and Eight Biopharmaceuticals Corp (Edison, NJ, USA)	I	NCT03486301
BDC-1001		Advanced Cancers	Not progressed	Bolt Biotherapeutics Inc. (Redwood, CA, USA)	I/II	NCT04278144
LHC-165	i.t	Advanced malignancies	Not reported	Novartis AG (Basel, Switzerland)	I	NCT03301896
SHR-2150	i.t	Unresectable/Metastatic Solid Tumors	Not reported	Chinese PLA General Hospital (Beijing, China)	I	NCT04588324
RO-7119929	Oral	Advanced primary or metastatic liver cancers	Identified dose-limiting safety risk	F. Hoffmann-La Roche Ltd. (Basel, Switzerland)	I	NCT04338685
	i.v	Advanced pancreatic adenocarcinoma	Disease control rate of 38% manageable safety profile	Institute Bergonie (Bordeaux Cedex, France)	II	NCT03915678
EIK1001	i.v	Stage 4 NSCLC	Evaluating the safety and efficacy	Eikon Therapeutics (Hayward, CA, USA)	II	NCT06246110
INI-4001	i.v	Advanced solid tumors	First-in-human trial Nanoparticle delivery system; study ongoing in Australia	Inimmune Corporation (Missoula, MT, USA)	I	NCT06302426
TransCon TLR7/8 Agonist	i.t	Advanced solid/metastasis tumors	Intratumoral delivery aims to enhance local immune response while reducing systemic exposure	Ascendis Pharma Oncology Division A/S (Palo Alto, CA, USA)	I/II	NCT04799054
BDB018		Advanced solid tumors	Designed for enhanced immune activation	Eikon Therapeutics	I	NCT04840394
NKTR-262	i.t	Relapsed/refractory metastatic melanoma	Enhanced systemic activation of T and NK cells with minimal toxicity	Nektar Therapeutics (San Francisco, CA, USA)	II	NCT03435640

## Data Availability

This review article does not contain any new data.

## Abbreviations

The following abbreviations are used in this manuscript:

TLRs	Toll-like receptors
PRRs	pattern recognition receptors
APCs	Antigen-presenting cells
DCs	dendritic cells
CTLs	cytotoxic T lymphocytes
NK	natural killer
IMQ	imiquimod
PAMPs	pathogen-associated molecular patterns
DAMPs	damage-associated molecular patterns
MyD88	Myeloid Differentiation Primary Response Gene 88
MDSCs	myeloid-derived suppressor cells
Tregs	regulatory T cells
NE	nanoemulsion
MOF	metal–organic framework
ICD	immunogenic cell death
BMDCs	bone marrow-derived dendritic cells
DOX	doxorubicin
TAA	tumor-associated antigen
NCs	nanocomposites
R848@NPs	R848-loaded PANi-conjugated glycol-chitosan nanoparticles
PARE NPs	prodrug-based nanoparticles
ARNPs	acidic-responsive polymeric nanoparticles
UCNPs	upconversion nanoparticles
PDT	Photodynamic therapy
RT	radiation therapy
SDT	Sonodynamic therapy
dLNs	draining lymph nodes
